# Mesenteric Abscess Complicated by Septic Peritonitis in a Dog: Multimodal Diagnosis, Surgical Management, and Serial Quantitative Bacterial Load Monitoring

**DOI:** 10.3390/vetsci13090946

**Published:** 2026-09-11

**Authors:** Daniel Schidu, Alina Levința, Paula Cucu, Codrin Moldovanu-Tonița, Eusebiu Viorel Șindilar, Liviu Cătălin Burtan, Vlad Zelinschi, Gheorghe Solcan

**Affiliations:** 1 Clinics Department, Faculty of Veterinary Medicine, “Ion Ionescu de la Brad” Iasi University of Life Sciences, M. Sadoveanu Alley 8, 700489 Iasi, Romania; schidu.daniel@gmail.com (D.S.); alina.levinta@iuls.ro (A.L.); eusebiu.sindilar@iuls.ro (E.V.Ș.); liviu.burtan@iuls.ro (L.C.B.); zelinschi.vlad96@gmail.com (V.Z.); 2 Department of Food Safety and Public Health, Faculty of Veterinary Medicine, “Ion Ionescu de la Brad” Iasi University of Life Sciences, M. Sadoveanu Alley 8, 700489 Iasi, Romania; paula.cucu@iuls.ro; 3 Department of Preclinical Sciences, Faculty of Veterinary Medicine, “Ion Ionescu de la Brad” Iasi University of Life Sciences, M. Sadoveanu Alley 8, 700489 Iasi, Romania; codrin.tonita@iuls.ro; 4 Academy of Agricultural and Forestry Sciences, 011646 Bucharest, Romania

**Keywords:** dog, mesenteric abscess, septic peritonitis, serial ultrasonography, quantitative bacterial culture, case report

## Abstract

Mesenteric abscesses are an uncommon cause of septic peritonitis in dogs, and serial quantitative monitoring of bacterial load in peritoneal exudate has rarely been reported. This case report describes the diagnostic work-up and treatment response of a 14-month-old, 33 kg intact male Cane Corso with a mesenteric abscess complicated by septic peritonitis, two weeks after treatment for babesiosis. The dog presented with a 72 h history of anorexia, vomiting, and abdominal pain. Abdominal radiography revealed decreased peritoneal serosal contrast and findings suggestive of foreign material ingestion. Serial ultrasonography identified a mesenteric abscess with peritoneal effusion. Exploratory laparotomy confirmed the diagnosis; the abscess was excised, and the abdominal lavage was performed. Histopathology confirmed a mesenteric abscess with marked neutrophilic infiltration. Bacterial culture isolated *Escherichia coli* and *Enterococcus faecium*, with antimicrobial therapy guided by susceptibility testing. Serial quantitative bacterial counts of the peritoneal exudate on three postoperative occasions showed a progressive decline, paralleling clinical and hematological improvement. The dog fully recovered, with complete clinical and laboratory normalization confirmed at the 60-day reassessment. This case suggests that a multimodal diagnostic approach may assist in guiding management and confirming treatment response in canine mesenteric abscess with septic peritonitis, although the role of serial quantitative monitoring requires further evaluation.

## Simple Summary

Mesenteric abscesses are rare in dogs and can lead to a life-threatening infection of the abdominal cavity called septic peritonitis. This report describes the case of a young dog that developed such an abscess after treatment for a blood parasite infection. The veterinary team used a combination of tools to diagnose and monitor the condition: X-rays, repeated ultrasound scans, laboratory blood tests, bacterial cultures with antibiotic sensitivity testing, and microscopic examination of the removed tissue. A distinctive feature of this report is that the bacteria in the abdominal fluid were counted three times after surgery, showing a steady decline that matched the dog’s clinical improvement. The abscess was surgically removed, the abdomen was washed, and antibiotics were chosen based on laboratory results. The dog recovered fully and was healthy at the one-month check-up. This case illustrates that combining imaging, bacterial counting, and tissue examination can support accurate diagnosis, treatment selection, and confirmation of recovery. As serial bacterial counting is rarely documented in veterinary medicine, it may complement clinical judgment in monitoring treatment response, though its ability to guide antimicrobial duration and drain removal requires further study.

## 1. Introduction

This case report was prepared in accordance with the CARE (CAse REport) reporting guidelines [1,2]. All relevant items of the CARE checklist have been reviewed and are addressed in the corresponding sections of this report. A chronological summary of the principal clinical events, interventions, and serial monitoring during the 11-day hospitalization is provided in Supplementary Table S1. Mesenteric lymph node abscessation and purulent lymphadenitis are uncommon but clinically important causes of acute abdominal disease in dogs and cats, and have been described almost exclusively as isolated case reports [3,4]. In the largest retrospective series to date, Salavati Schmitz identified only 14 dogs with idiopathic purulent mesenteric lymphadenitis or lymph node abscessation over a ten-year period at a single teaching hospital, and no primary underlying cause was identified in any case [5]. Reported bacterial isolates from canine and feline mesenteric lymphadenitis and abscessation include *Escherichia coli*, *Staphylococcus* spp., and, rarely, *Salmonella* spp. [3,4,5,6], a pattern that closely parallels the predominance of Enterobacteriaceae, streptococci, and staphylococci reported in larger surveys of septic peritonitis in dogs, cats, and other domestic species [6,7].

When a mesenteric abscess ruptures or otherwise contaminates the peritoneal cavity, the result is secondary septic peritonitis, the most common and most life-threatening form of peritonitis encountered in small animal practice [8,9,10]. Despite advances in surgical source control, broad-spectrum antimicrobial therapy, and perioperative critical care, survival in dogs with septic peritonitis remains guarded and highly variable; recent reviews summarize survival rates ranging from 36.4% to 88.5% for gastrointestinal-source septic peritonitis [8,9]. In a recent retrospective study of 161 dogs, survival to discharge was 70.8%, with nonsurvival associated with older age, lower total plasma protein and serum albumin concentrations, and lower systolic blood pressure at presentation, whereas admission lactate was not significantly associated with survival in that cohort [11]. Because clinical signs such as inappetence, vomiting, abdominal pain, and fever are frequently nonspecific, cytological confirmation of septic effusion and, increasingly, biomarker-guided testing are recommended to support timely diagnosis [6,10,12].

Serial abdominal ultrasonography is central to both the initial diagnosis of intra-abdominal abscessation or effusion and to postoperative monitoring, although its sensitivity for correctly identifying the underlying lesion in septic peritonitis remains imperfect, ranging from approximately 56% to 87% across recent series [13,14,15]. In contrast, direct quantitative monitoring of bacterial load within the peritoneal cavity during recovery has rarely been reported in veterinary medicine; only a small number of studies have evaluated sequential bacterial culture as an objective marker of treatment response after surgical and antimicrobial management of septic peritonitis [12,16].

Here we report a young Cane Corso dog that developed a mesenteric abscess complicated by septic peritonitis in the weeks following treatment for babesiosis, with radiographic findings suggestive of foreign material ingestion as a possible inciting factor. *Escherichia coli* and *Enterococcus faecium* were isolated from the abdominal effusion, consistent with previously reported etiologies of canine septic peritonitis [6,7]. To our knowledge, this is among the few reports to document serial quantitative, rather than purely qualitative, bacterial load monitoring over the postoperative period, together with serial ultrasonographic assessment, in a dog recovering from mesenteric abscess-associated septic peritonitis. The objective of this case report is to describe the clinical presentation, diagnostic imaging, surgical and antimicrobial management, and quantitative postoperative bacterial monitoring in this dog, and to highlight the potential diagnostic value of this monitoring approach. As a single case with a favorable outcome, this report cannot establish prognostic utility; the parallel decline in bacterial counts and clinical improvement observed in this patient is therefore presented as preliminary clinical evidence rather than as a demonstration of prognostic value.

## 2. Case Description

A 14-month-old male intact Cane Corso dog, weighing 33 kg and outdoor-housed with up-to-date vaccination and deworming, was presented with a chief complaint of anorexia, vomiting, and abdominal pain. Clinical signs had been present for approximately 72 h at presentation, superimposed on approximately 10 days of intermittent inappetence following treatment for babesiosis. Babesiosis had been diagnosed two weeks earlier at a referring veterinary practice on the basis of a peripheral blood smear and treated with imidocarb dipropionate (Imizol), after which the dog developed marked generalized weakness. On presentation to our hospital, a repeat peripheral blood smear was negative for *Babesia* organisms, indicating resolution of parasitemia by the time of abscess presentation; the marked thrombocytopenia observed at admission was therefore attributed to the septic process rather than to active babesiosis. On physical examination, temperature was 39.5 °C, heart rate was 100 bpm, and respiratory rate was 40 breaths/min. This temperature lies at the upper limit of the recently established reference interval for rectal temperature in adult dogs (37.7–39.5 °C) [17] and is consistent with a low-grade fever, since larger dogs have been shown to have lower resting rectal temperatures than smaller dogs [18], a consideration particularly relevant for a large-breed Cane Corso. The dog was responsive but subdued and less alert than normal, with marked generalized weakness and severe loss of body condition. Mucous membranes were pink with a capillary refill time under 2 s. Abdominal palpation elicited pain; no peripheral lymphadenopathy was palpable, and rectal examination revealed feces of normal color and consistency.

Initial complete blood count revealed leukocytosis with neutrophilia (WBC 26.6 × 10^9^/L, reference interval 6–17 × 10^9^/L; neutrophils 23.73 × 10^9^/L, reference interval 3–12 × 10^9^/L), mild anemia (RBC 4.59 × 10^12^/L, hemoglobin 9.9 g/dL, hematocrit 32.51%), and marked thrombocytopenia (52 × 10^9^/L, reference interval 165–500 × 10^9^/L). Initial serum biochemistry showed markedly elevated ASAT (197 U/L) and alkaline phosphatase (504 U/L), with ALT within reference range (89 U/L); hypoproteinemia (52 g/L) and hypoalbuminemia (22 g/L); elevated creatine phosphokinase (685 U/L); hypocalcemia (total calcium 6.96 mg/dL, ionized calcium 1.14 mmol/L), hypomagnesemia (0.74 mg/dL), and hypokalemia (2.22 mmol/L); and mild hyponatremia (131 mmol/L), together with mildly elevated total and direct bilirubin (0.42 and 0.11 mg/dL, respectively). Hematological analyses were performed using a Mindray BC-5300Vet hematology analyzer (Mindray, Shenzhen, China) with manufacturer-provided reference intervals, and serum biochemistry using a BioSystems BA200 analyzer (BioSystems, Barcelona, Spain); the biochemical reference intervals used were those of the Merck Veterinary Manual [19] (ASAT 13–15 U/L; ALP 1–114 U/L; ALT 10–109 U/L; creatine kinase 52–368 U/L; total protein 54–75 g/L; albumin 23–31 g/L; total calcium 9.1–11.7 mg/dL; magnesium 1.6–2.4 mg/dL; potassium 3.9–5.1 mmol/L; sodium 142–152 mmol/L; total bilirubin 0–0.3 mg/dL), supplemented by published reference intervals for ionized calcium (1.18–1.37 mmol/L [20]) and direct bilirubin (0–0.1 mg/dL [21]).

**Figure 1:**
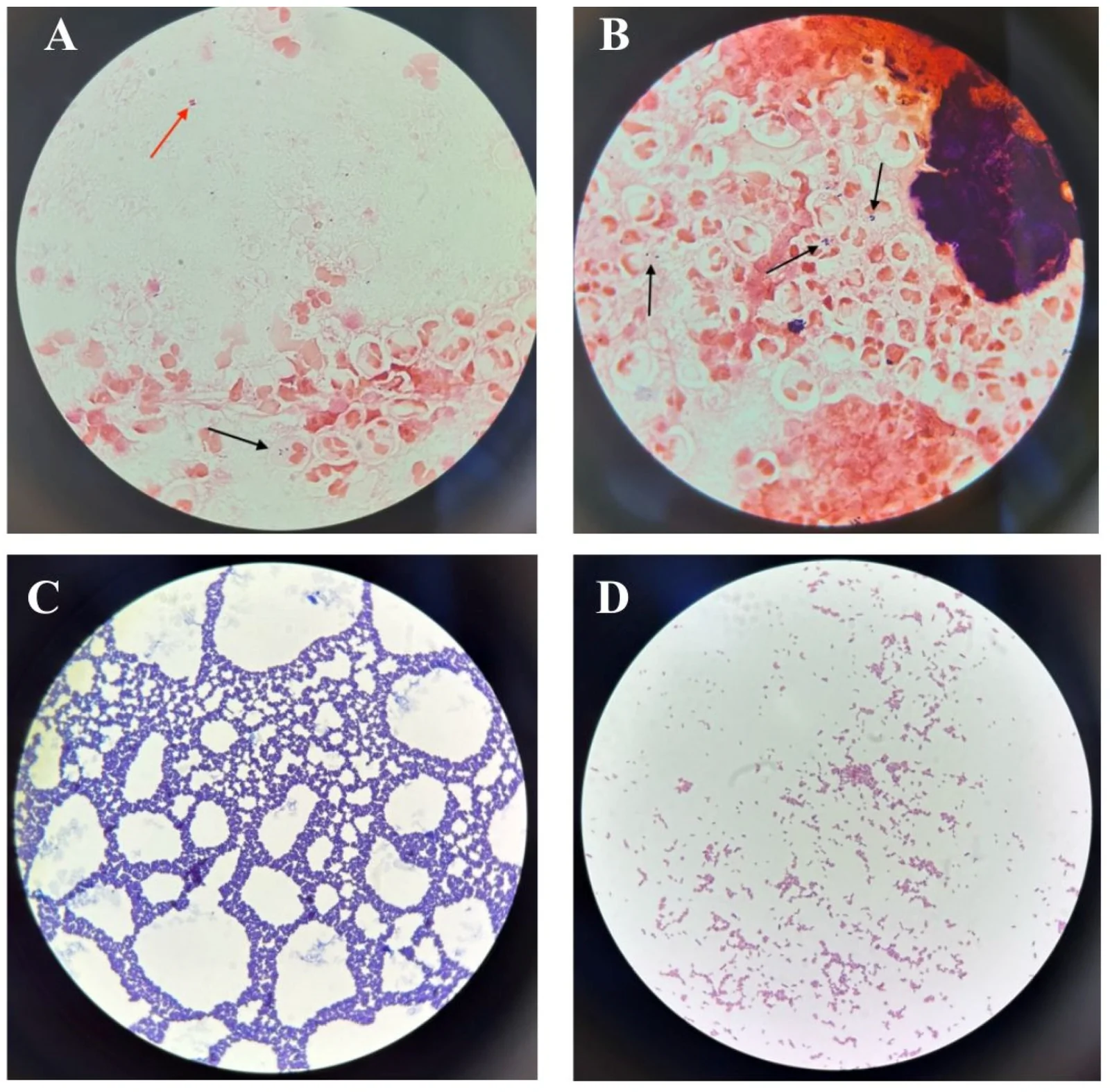
Direct microscopic examination of peritoneal fluid/abscess content by Gram stain. (**A**) Gram stain, × 1000: Gram-negative bacilli (red arrow) and phagocytosed Gram-positive cocci (black arrow). (**B**) Gram stain, × 1000: Gram-positive cocci and numerous polymorphonuclear cells. (**C**) *Enterococcus faecium*, microscopic examination of a pure culture, Gram stain, × 1000 magnification. (**D**) *Escherichia coli*, microscopic examination of a pure culture, Gram stain, × 1000 magnification.

Bacteriological culture and direct microscopic examination of a Gram-stained smear were performed on peritoneal fluid/abscess content collected during laparotomy. Gram staining showed a septic suppurative exudate with numerous polymorphonuclear cells and mixed bacterial morphologies, including Gram-negative bacilli and Gram-positive cocci, with phagocytosed Gram-positive cocci observed (Figure 1). Peritoneal-fluid biochemical analysis was not performed. Plasma lactate concentration was within the reference interval on both occasions on which it was measured (7.3 and 18.7 mg/dL; reference interval 1.8–22.5 mg/dL); blood gas analysis and a coagulation profile were not performed.

## 3. Results

### 3.1. Clinical Course and Laboratory Monitoring

The clinical course over the 11-day hospitalization is summarized here to contextualize the diagnostic and therapeutic findings detailed below. At presentation (Day 1), the dog exhibited abdominal pain, lethargy, vomiting, and reduced appetite (temperature 39.5 °C, heart rate 100 bpm, respiratory rate 40/min, pink mucous membranes, capillary refill time under 2 s, abdominal pain on palpation); physical examination and fluid therapy were initiated together with butylscopolamine (Buscopan^®^, hyoscine butylbromide, 0.3 mg/kg IV every 12 h for 4 days), metronidazole (Metronidazole 5 mg/mL, 100 mL, B. Braun, 15 mg/kg IV every 12 h for 4 days), and amoxicillin-clavulanate (Synulox^®^, 20 mg/kg SC every 24 h for 4 days). By Day 2, the dog remained ambulatory but inappetent (temperature 40.8 °C, heart rate 108 bpm, respiratory rate 52/min); abdominal ultrasonography was followed by exploratory laparotomy, during which peritoneal lavage and complete resection of a mesenteric abscess (which had already spontaneously drained into the abdominal cavity) were performed, and samples were collected from the adherent mass and abscess contents; fluid therapy with Ringer’s solution (Ringer Fresenius, 3 mL/kg/h, a continuous maintenance rate for the first 4 days), Synulox, and butylscopolamine were continued, and tranexamic acid (Ugurol^®^, 15 mg/kg IV every 12 h for 3 days) was added. Tranexamic acid was administered prophylactically, given the marked thrombocytopenia (52 × 10^9^/L) and mild anemia documented at admission, to minimize intraoperative hemorrhage and reduce the risk of postoperative microhemorrhage [22]. Postoperative analgesia was provided with methadone (Insistor Vet^®^, 0.4 mg/kg IV every 8 h during the first 48 h postoperatively, followed by 0.25 mg/kg every 12 h for the following 4 days) and meloxicam (Melovem^®^ 5 mg/mL, 0.2 mg/kg SC on the first postoperative day, followed by 0.1 mg/kg for 3 days). Only on Day 3 did the patient have a stable temperature of 38.6–38.8 °C, fluctuating appetite, two stools of normal consistency, and one diarrheic stool at 6:00 PM. On Day 4, the dog was still ambulatory with reduced appetite; bacterial culture and susceptibility results became available (*Escherichia coli,* sensitive to enrofloxacin; *Enterococcus faecium,* sensitive to chloramphenicol), prompting adjustment of antimicrobial therapy to enrofloxacin (Enroxil^®^, 5 mg/kg SC for 7 days from the time susceptibility results were obtained) and chloramphenicol (CTP^®^, 20 mg/kg SC every 12 h for 7 days from the time susceptibility results were obtained). By Day 5, appetite had returned, and the dog was normothermic (temperature 38.8 °C, heart rate 90 bpm, respiratory rate 32/min), and the susceptibility-guided antimicrobial course was continued for a total of 7 days. The dog was discharged on Day 11, clinically stable following completion of this course. No antimicrobial medication was prescribed for home administration after discharge; the susceptibility-guided course was completed during hospitalization. In total, antimicrobial therapy comprised an 11-day course: a 4-day empirical phase (amoxicillin–clavulanate plus metronidazole, from admission on Day 1 until susceptibility results on Day 4, spanning the day before surgery and the first three postoperative days) followed by a 7-day susceptibility-guided phase (enrofloxacin plus chloramphenicol, Days 4–11), completed at discharge.

Serial laboratory monitoring throughout hospitalization complemented this clinical course. Complete blood count, assessed on nine occasions, showed progression from the leukocytosis with neutrophilia and marked thrombocytopenia noted at presentation (see Section 2) to a peak white blood cell count of 56.09 × 10^9^/L, with the initial anemia partially recovering and platelet count progressively normalizing from 52 × 10^9^/L to 469 × 10^9^/L. The WBC peak occurred at 24 h postoperatively (POD1), at which point the patient was already normothermic (38.6–38.8 °C) with no recurrence of abdominal pain; this marked postoperative leukocytosis is most consistent with the inflammatory leukocyte response to the septic abdomen and surgical tissue trauma, compounded by endogenous stress-induced demargination of neutrophils (catecholamine and cortisol surge) [23], rather than aggravation of infection—which is further supported by the concurrent decline in quantitative bacterial load (POD1–POD3) and by the subsequent fall in WBC as the patient improved. A drug reaction is unlikely, as none of the administered agents are associated with leukocytosis, and chloramphenicol in particular causes bone marrow suppression, with the WBC declining during its administration. Serum biochemistry, assessed on five occasions using a serum biochemistry analyzer (BioSystems BA200, Barcelona, Spain), showed progressive normalization of the markedly elevated liver enzymes (ASAT, ALT, and alkaline phosphatase) noted at presentation, persistent hypoproteinemia and hypoalbuminemia, elevated creatine phosphokinase, and progressive correction of the initial hypocalcemia, hypomagnesemia, and hypokalemia, together with resolution of the mild hyponatremia.

### 3.2. Imaging Diagnosis

**Figure 2:**
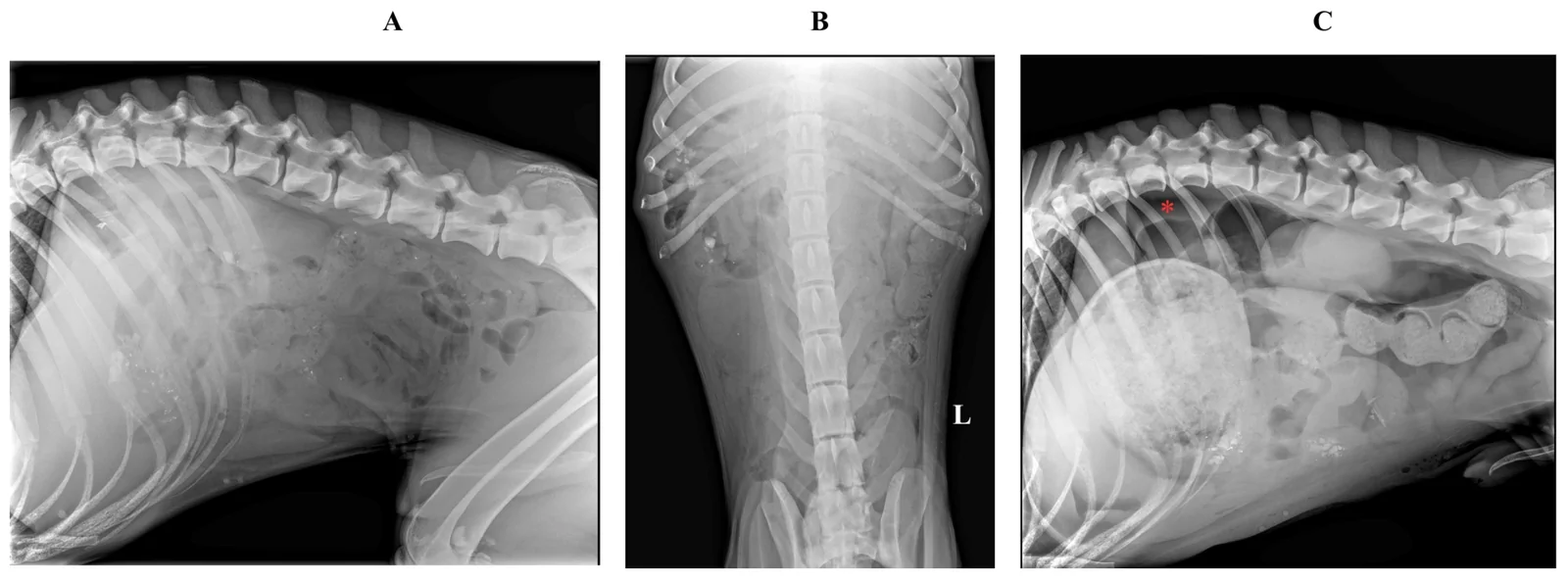
Abdominal radiographs of the patient. (**A**) Right lateral view at presentation. (**B**) Dorsoventral view at presentation. (**C**) Right lateral view at three days postoperatively.* *Pneumoperitoneum.*

Abdominal radiography (at presentation, Figure 2A,B): peritoneal contrast was decreased, with poor delineation of intestinal loops and moderate intestinal distension. The gastric silhouette was distended, containing multiple hard/mineral radiopacities and a discernible bone radiopacity on the left lateral view, and the pylorus showed moderate distension with fluid content, consistent with foreign material ingestion. No foreign body was, however, identified or removed during laparotomy, and these gastric radiopacities most likely represented ingested food or mineral material rather than a persistent foreign body. The suspicion of foreign material ingestion was therefore not confirmed surgically or histopathologically, and its contribution to the formation of the mesenteric abscess remains speculative; the precise origin of the mesenteric abscess could not be established: no foreign body was identified at surgery or histopathology, and no communication between the intestinal lumen and the abscess cavity was demonstrated, so the mechanism of abscess formation remains speculative. These findings raised suspicion of a peritoneal reaction with a small volume of free fluid; abdominal ultrasonography was recommended to further assess peristalsis and the appearance of the peritoneal fat. At three days postoperatively, repeat abdominal radiography (Figure 2C) showed increased peritoneal contrast relative to the initial examination, retroperitoneal pneumoperitoneum, and well-delineated gastrointestinal content and intestinal loops.

**Figure 3:**
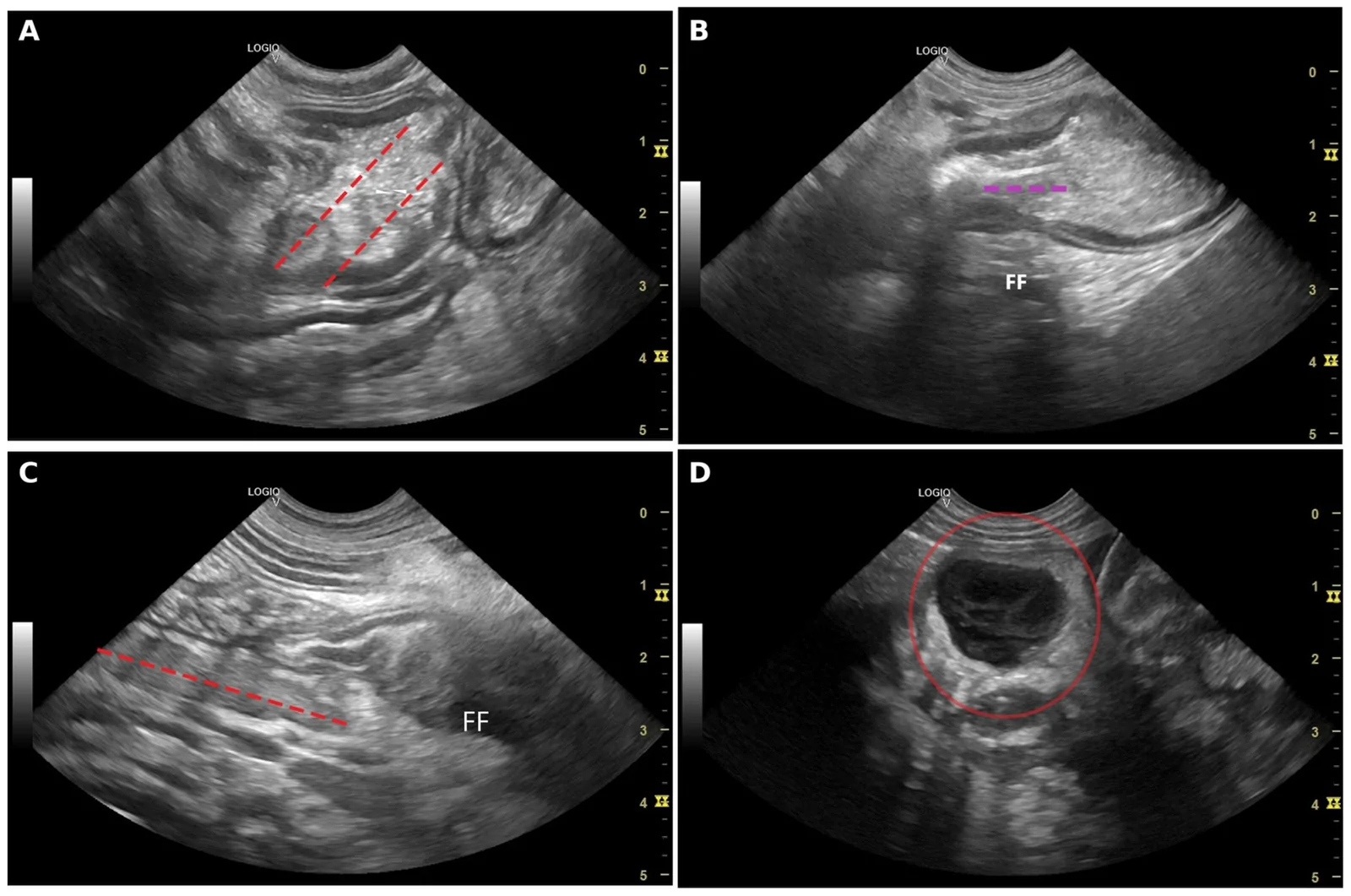
Abdominal ultrasonography findings at presentation. (**A**) Distended (marked with two red dashed lines), fluid-filled small intestinal loops with accelerated peristalsis. (**B**) Wall with normal stratification and thickness; jejunal lumen severely distended with mixed, heterogeneous content; intraluminally, a linear-appearing structure is observed (purple dashed line), hyperechoic relative to the intestinal content, without posterior acoustic shadowing. (**C**) Enlarged mesenteric lymph node (red dashed line) with normal echostructure and echogenicity, consistent with reactive lymphadenopathy. (**D**) Circular/ovoid structure with predominantly anechoic content and hypoechoic conglomerates in suspension (red circle), raising suspicion of an abscess. FF, free fluid.

Abdominal ultrasonography (Figure 3): the small intestinal loops were diffusely distended, with accelerated peristalsis; on dynamic assessment, a topographic discontinuity of the jejunal loops was identified (suggestive of apparent intestinal occlusion), although complete visualization was precluded by artifacts. A linear, markedly echogenic structure was also noted. The dog exhibited a focal pain response when the probe was placed over the mid-abdomen. Endoscopic examination and/or exploratory laparotomy were recommended.

The stomach was distended, with a heterogeneous muscular layer, thickened mucosa, and accelerated gastric peristalsis; content was mixed, predominantly anechoic and gaseous, precluding complete evaluation, and the pyloric portion was not visualized. The liver had an apparently normal echostructure, echogenicity, and size, and the gallbladder wall was thin and hyperechoic with anechoic content. The spleen was enlarged, with normal echostructure and echogenicity but rounded lobar margins extending beyond its normal anatomical projection. The descending colon was distended with mixed content; wall stratification and thickness were otherwise unremarkable.

In the small intestine, severe luminal dilation with “to-and-fro” peristalsis and mixed, heterogeneous content was observed in the cranial and mid-abdomen; the intestinal mucosa was heterogeneous, with segments of differing caliber, and peristalsis was accelerated. Within a dilated jejunal loop, a linear, hyperechoic structure with “dirty” posterior acoustic shadowing was identified. In the right mid-abdomen, at the anatomical projection of the ileocecal valve, the complete course of the intestinal loops could not be traced, consistent with intestinal occlusion. A small amount of anechoic free fluid was diffusely distributed among the small intestinal loops, not amenable to aspiration, together with an adjacent peritoneal reaction. Mesenteric lymph nodes were enlarged, with normal echostructure and echogenicity, consistent with reactive lymphadenopathy. A circular/ovoid structure with predominantly anechoic content and hypoechoic conglomerates in suspension was also identified, raising suspicion of an abscess. Suspected cystitis was noted on evaluation of the pelvic region; the urinary bladder was in a semi-distended state, with a thickened wall (approximately 0.59 cm) and anechoic content.

### 3.3. Antimicrobial Susceptibility Testing

Antimicrobial susceptibility testing (AST) showed a mixed bacterial infection with *Escherichia coli* and *Enterococcus faecium* isolated from intraoperatively collected peritoneal fluid and abscess content. Because the volume of free peritoneal fluid identified ultrasonographically was small and diffusely distributed among the intestinal loops, and thus not amenable to safe percutaneous aspiration, diagnostic abdominal puncture was not performed; bacteriological samples for culture, susceptibility testing, and Gram-stain cytology were instead obtained intraoperatively during exploratory laparotomy, whereas serial quantitative bacterial monitoring was performed on peritoneal exudate collected through the abdominal drain during the first three postoperative days (POD1–POD3). Direct Gram stain of the sample revealed Gram-positive cocci arranged in pairs and chains, Gram-negative bacilli, and occasional Gram-positive bacilli. Bacterial identification was performed using the MALDI-TOF system (Zybio EXS 2600), and AST interpretation followed the CLSI VET01 standard (2025 edition), with veterinary-specific interpretive criteria (clinical breakpoints) applied for both *Escherichia coli* and *Enterococcus faecium*; no human (CLSI M100) breakpoints or extrapolated criteria were used for susceptibility interpretation.

For *Escherichia coli*, the isolate was susceptible to enrofloxacin and meropenem and resistant to ampicillin, amoxicillin–clavulanate, cefuroxime, doxycycline, chloramphenicol, and trimethoprim–sulfamethoxazole.

For *Enterococcus faecium*, the isolate was susceptible only to chloramphenicol and resistant to ampicillin, doxycycline, vancomycin, and enrofloxacin.

### 3.4. Quantitative Bacterial Monitoring

The bacterial load in the peritoneal fluid was quantified by determining the total germ count (TGC), using the serial decimal dilution method followed by inoculation on solid media, according to the formula TGC = N/(V × d), at three successive time points (POD1, POD2, and POD3).

where
**Symbol****Definition**TGCTotal germ count (CFU/mL of original peritoneal fluid)NNumber of colonies counted on the selected culture plateVVolume of inoculum plated on solid medium (mL)dDecimal dilution factor of the plated inoculum

For each time point, two serial decimal dilutions (10⁻^2^ and 10⁻^3^) were plated in parallel; 100 µL (0.1 mL) of each dilution was inoculated onto solid media and incubated at 36 °C for 24 h, and colonies were counted on plates yielding 30–300 colonies. Samples were plated within one hour of collection to minimize pre-analytical changes in viable counts. The two dilution-based counts obtained for each sample were concordant and of the same order of magnitude, indicating good reproducibility of the method; the range reported for the first two time points therefore represents the two independent counts from the two dilutions of the same sample, while for the third time point (POD3) only the 10⁻^2^ dilution yielded countable growth and a single value is reported. To standardize sampling and allow comparison across time points, all three quantitative cultures were obtained from the abdominal drainage tube and collected once daily (approximately 24 h apart) before routine drain care; the drain was not irrigated prior to sampling, so the analyzed effluent represented spontaneous drainage. Surgery was performed on hospital Day 2, so the three collections correspond to Days 3, 4, and 5 (POD1, POD2, and POD3). The second collection (POD2/Day 4) coincided with the return of susceptibility results and the switch from empirical therapy (amoxicillin–clavulanate plus metronidazole) to susceptibility-guided therapy (enrofloxacin plus chloramphenicol); the third collection (POD3/Day 5) was obtained after this change. The drain was maintained for the first three postoperative days and removed after the POD3 collection, once serial counts had shown a consistent decline. Serial decimal dilutions were prepared in sterile 0.9% saline, beginning with 1 mL of peritoneal fluid diluted 1:10 (1 mL sample plus 9 mL diluent) to yield the 10⁻^1^ dilution and continuing with serial 1:10 transfers for the 10⁻^2^ and 10⁻^3^ dilutions; the inoculated medium was Tryptic Soy Agar (TSA), and the reported counts represent the total cultivable aerobic bacterial population (mixed *Escherichia coli* and *Enterococcus faecium*) rather than species-specific counts, species identification having been performed separately by MALDI-TOF mass spectrometry on pure subcultures.

At the first sampling (POD1), TGC values were 3.12 × 10^5^–4.3 × 10^5^ CFU/mL, indicating a high initial bacterial load, consistent with a severe intra-abdominal infectious process. At the second sampling (POD2), TGC values were 2.31 × 10^5^–2.6 × 10^5^ CFU/mL, showing a decreasing trend and an early favorable microbiological response to the antimicrobial treatment instituted according to the antibiogram. At the third sampling (POD3), TGC decreased to 2.8 × 10^4^ CFU/mL, reflecting a marked reduction in peritoneal bacterial contamination, consistent with the patient’s favorable clinical progress. However, because surgical source control, abscess drainage, peritoneal lavage, drain placement, and antimicrobial therapy were undertaken concurrently, the decline in bacterial burden cannot be attributed specifically to the change to enrofloxacin and chloramphenicol.

**Figure 4:**
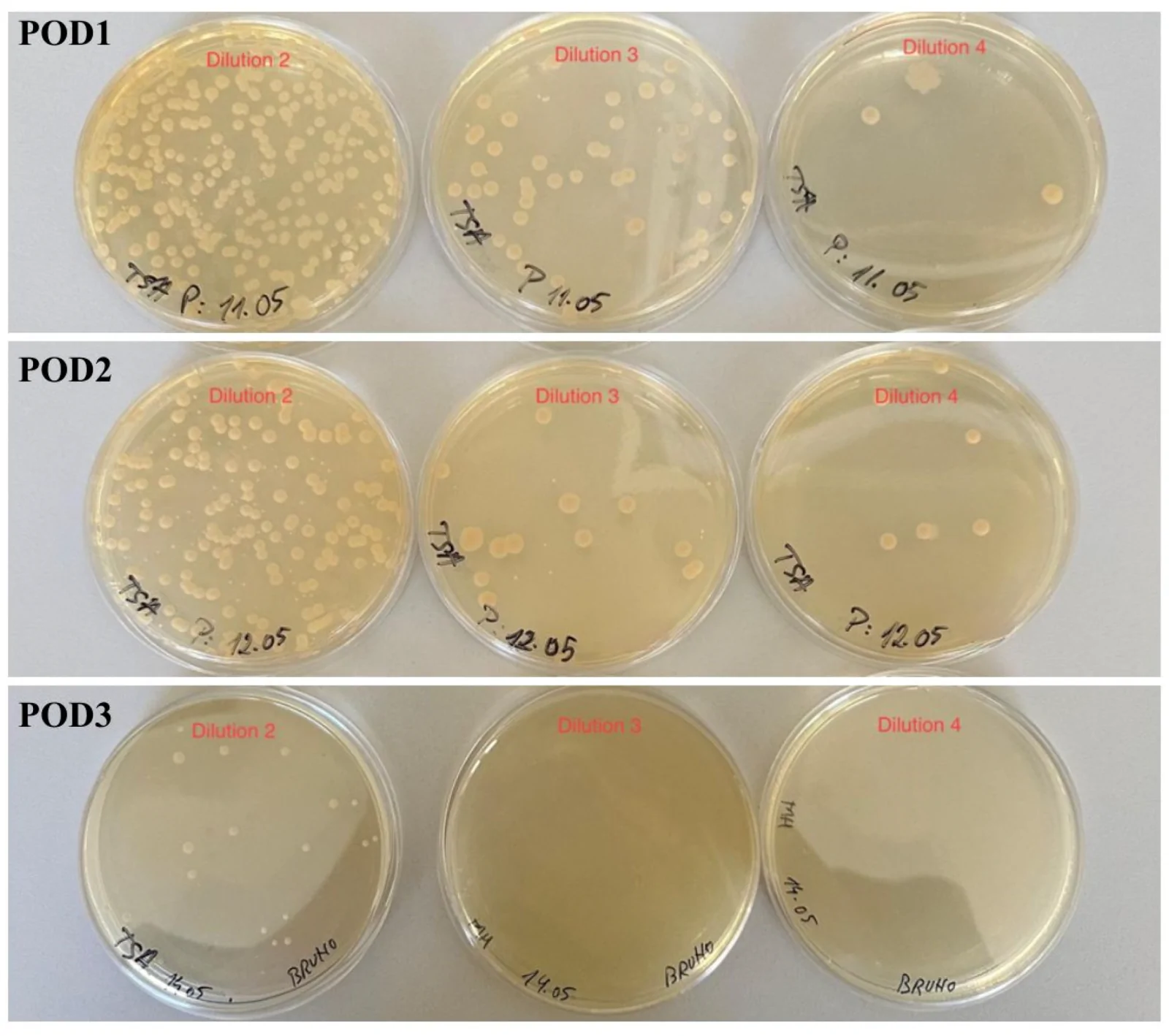
Quantitative bacterial monitoring—colony appearance on TSA medium. POD1 (postoperative day 1). TGC: 3.12–4.3 × 10^5^ CFU/mL. Mixed aerobic growth (Gram-positive cocci in pairs/chains; rare Gram-negative and Gram-positive bacilli). Drain sample collected via abdominal drain. POD2 (postoperative day 2). TGC: 2.31–2.6 × 10^5^ CFU/mL. Mixed aerobic growth. Susceptibility-guided antimicrobial therapy ongoing. POD3 (postoperative day 3). TGC: 2.8 × 10^4^ CFU/mL. Mixed aerobic growth. Progressive decline in bacterial count.

The macroscopic appearance of the colonies developed on TSA medium at the three samplings is shown in Figure 4.

### 3.5. Macroscopic Appearance and Surgical Treatment

**Figure 5:**
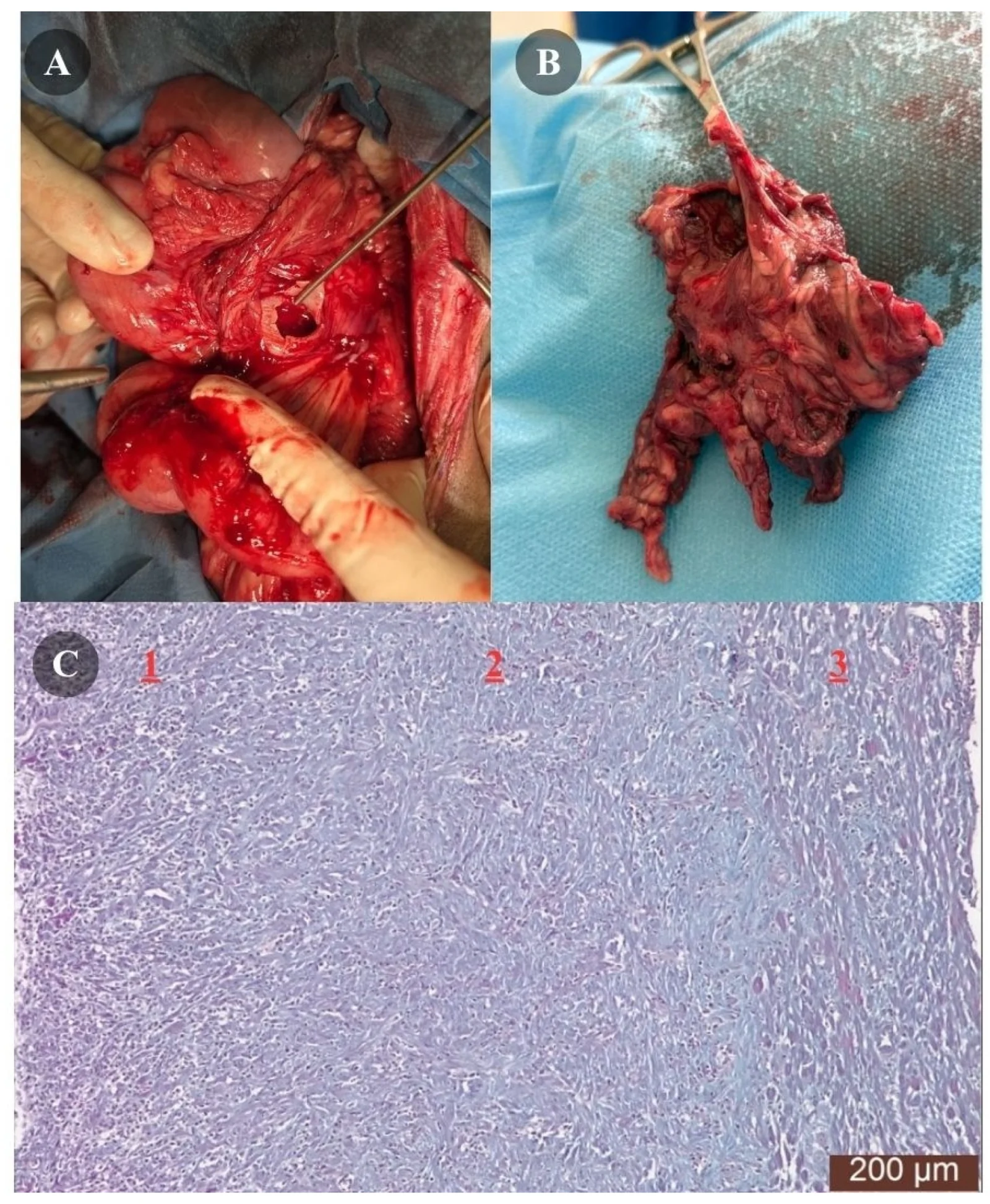
Intraoperative and histopathological findings. (**A**) Intraoperative view showing the fistulized abscess draining purulent content into the abdominal cavity. (**B**) Resected mesenteric abscess following en bloc excision. (**C**) Photomicrograph illustrating the three-layered histological architecture of the abscess wall (hematoxylin–eosin–azure staining; scale bar = 200 µm): (1) inner zone; (2) intermediate zone; (3) outer zone.

Intraoperatively, an adherent intestinal block was identified, together with a peritoneal reaction characterized by the accumulation of purulent exudate and serohemorrhagic fluid. The parietal peritoneum displayed multiple ecchymoses, and the mesentery exhibited hematomas of variable size. Peristalsis was present and exaggerated along the entire length of the small intestine. Macroscopic changes were also noted at the level of the pancreas, which appeared enlarged in volume, together with splenic infarcts and gas-distended intestinal loops. Within the adherent block, a fistulized mass was identified, from which purulent content was draining into the abdominal cavity (Figure 5A).

**Figure 6:**
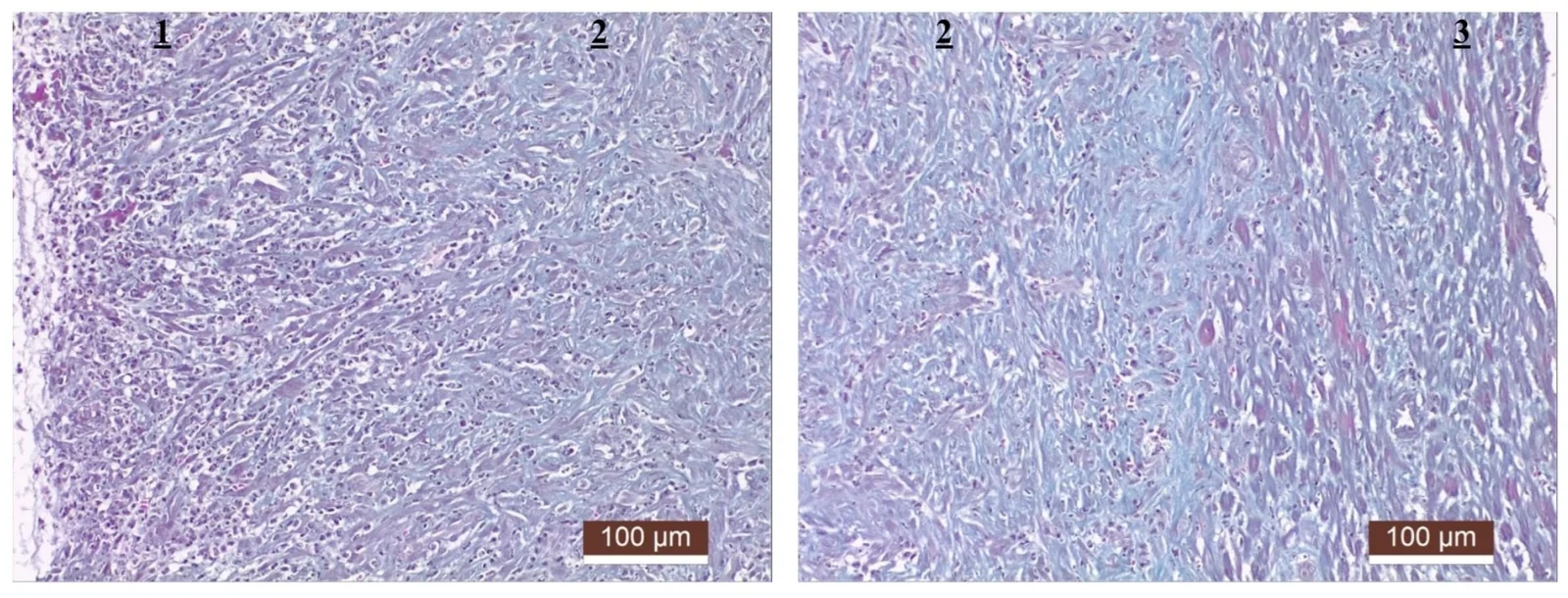
Higher-magnification photomicrograph illustrating the three-layered histological architecture of the abscess wall (hematoxylin–eosin–azure staining; scale bar = 100 µm): (1) inner zone; (2) intermediate zone; (3) outer zone.

During laparotomy, the fistulized abscess draining purulent content into the peritoneal cavity was identified within the adherent intestinal block (Figure 5A). Following careful adhesiolysis to free the adherent intestinal loops, the mesenteric abscess was removed en bloc (Figure 5B). The ultrasonographic suspicion of intestinal occlusion was therefore consistent with the surgical findings: the apparent topographic discontinuity of the jejunal loops reflected not only displacement and compression of adjacent loops by the mesenteric abscess within the adherent intestinal block, but also the associated peritoneal inflammatory reaction—with reflex ileus, intestinal wall thickening, adhesions, and loculated fluid and gas further distorting the topographic appearance of the loops—rather than a primary luminal obstruction; it resolved intraoperatively once the mesenteric abscess was removed and the adherent intestinal loops were freed by adhesiolysis. The intestine itself was not resected, intestinal peristalsis was preserved, and no intestinal anastomosis was required, with an uneventful recovery. Macroscopic inspection of the intestine during laparotomy revealed no evidence of perforation, necrosis, or ulceration, and no communication between the intestinal lumen and the abscess cavity was identified; the mesenteric abscess was a discrete structure within the mesentery that was fistulized into the peritoneal cavity. As the intestine was not resected, histopathological examination of the intestinal wall was not performed. The peritoneal cavity was then thoroughly lavaged with 200 mL/kg of 0.9% NaCl to reduce bacterial contamination and remove residual debris. An abdominal drain tube was placed and maintained during the first 3 postoperative days, through which peritoneal exudate was also collected for microbiological monitoring. The drain was managed as a passive closed-drainage system for evacuation of residual peritoneal fluid and serial sampling; no intermittent postoperative lavage was performed through the drain, both because intraoperative lavage had already addressed gross contamination and to avoid diluting the serial quantitative cultures. The resected specimen was subsequently submitted for histopathological examination (Figure 5C and Figure 6).

### 3.6. Histopathological Diagnosis

The tissue samples were stained using the hematoxylin–eosin–azure (HEA) protocol and subsequently subjected to histopathological examination. No special connective tissue stains (e.g., Masson’s trichrome) were performed. Microscopic evaluation confirmed the characteristic three-layered architecture of the abscess wall (Figure 5C), consisting of the following zones:

Inner zone, characterized by a dense population of activated neutrophils and macrophages exhibiting marked phagocytic activity, evidenced by prominent intracytoplasmic vacuolation, accompanied by a sparse lymphocytic infiltrate.

Intermediate zone, composed of lymphocytic infiltrates, proliferating fibroblasts, and collagen deposition, with scattered macrophages. The dense fibrovascular connective tissue is consistent with organized fibrous connective tissue; because only HEA staining was performed, the activity and maturity of the fibrotic process cannot be further characterized, and these observations are reported descriptively.

Outer zone, characterized by extensive fibrosis and the formation of a collagenous capsule. Higher-magnification micrographs illustrating the principal cellular populations and morphological features of each zone are presented in Figure 6.

### 3.7. Follow-Up and Outcomes

**Figure 7:**
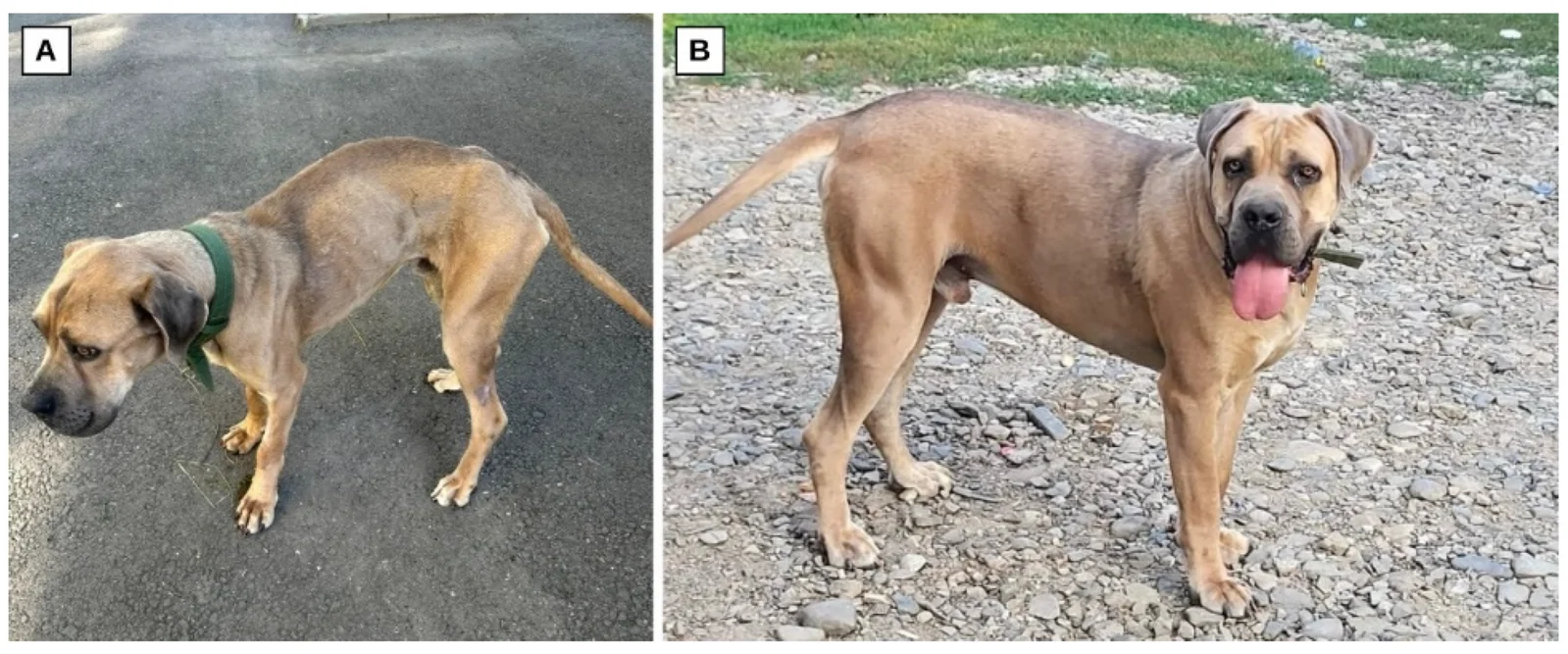
Clinical recovery documented by body weight change. (**A**) First day of hospitalization (33 kg). (**B**) Sixty days postoperatively (39 kg).

**Figure 8:**
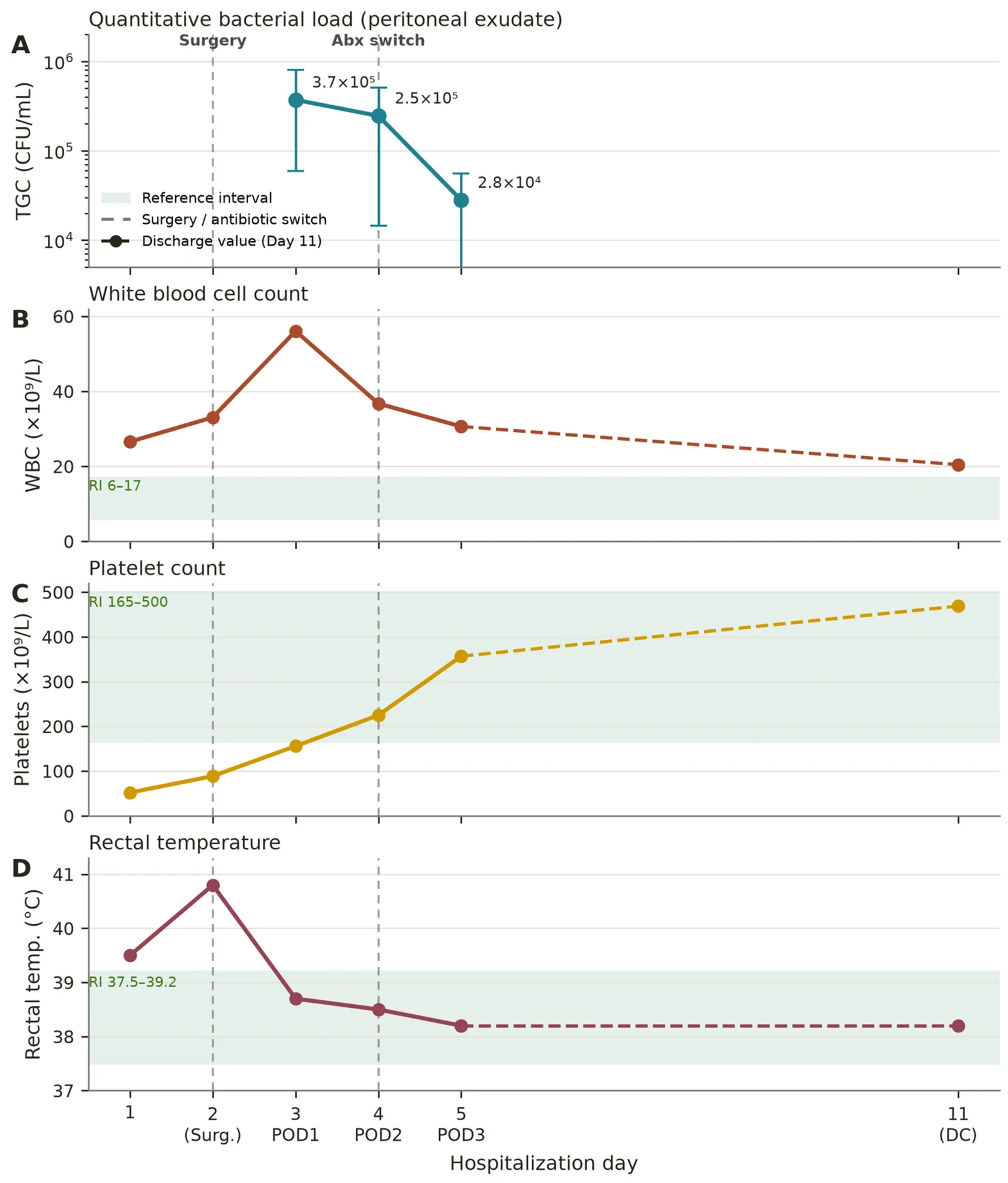
Integrated trend of clinical, laboratory, and bacteriological parameters over the 11-day hospitalization. (**A**) Quantitative bacterial load (TGC) of peritoneal exudate (logarithmic scale; error bars indicate the range between the two serial dilution counts, where available); (**B**) white blood cell count (shaded band = reference interval 6–17 × 10^9^/L); (**C**) platelet count (shaded band = reference interval 165–500 × 10^9^/L); (**D**) rectal temperature (shaded band = reference interval 37.5–39.2 °C). Dashed vertical lines indicate the day of exploratory laparotomy (Day 2) and the switch to susceptibility-guided antimicrobial therapy (Day 4). Open symbols connected by dashed lines denote the discharge value (Day 11). POD, postoperative day; DC, discharge.

Over the remainder of hospitalization, the clinical course was marked by progressive improvement, with gradual amelioration of clinical signs, normalization of vital parameters, and a stepwise return of appetite. Between Day 4 and Day 6, the dog developed diarrhea, which was managed with the introduction of a probiotic; stool consistency progressively normalized thereafter, and the probiotic was continued for a further 15 days after discharge. Serial hematological and biochemical monitoring performed throughout hospitalization showed a trend of progressive normalization that paralleled the decline in quantitative peritoneal bacterial counts (TGC), from approximately 3–4 × 10^5^ CFU/mL to 2.8 × 10^4^ CFU/mL across the three postoperative sampling occasions. Serial abdominal ultrasonographic reevaluations, performed at 2–3-day intervals, demonstrated progressive normalization of intestinal transit and gradual disappearance of the previously observed free peritoneal fluid. At the postoperative day 5 assessment, the small intestinal loops showed reduced luminal distension and normalization of peristalsis with resolution of the previously documented invagination tendency, and the volume of free peritoneal fluid was reduced compared with earlier evaluations. Following completion of the 7-day targeted antimicrobial course, the dog was discharged on Day 11 in clinically stable condition, with normal appetite and vital parameters, and was maintained on the probiotic regimen for a further 15 days at home. At the one-month follow-up examination, the dog was clinically normal, with no evidence of recurrence. A formal clinical reassessment at 60 days postoperatively confirmed complete recovery. The surgical wound was fully cicatrized, abdominal palpation elicited no sensitivity, appetite was present, and the patient was bright and alert. Body weight had increased from 33 kg at admission to 39 kg, with recovery of body condition (Figure 7). Hematology showed resolution of the previously documented leukocytosis (white blood cell count 13.57 × 10^9^/L; reference range 6–17 × 10^9^/L) and thrombocytopenia (platelets 450 × 10^9^/L; reference range 165–500 × 10^9^/L). Biochemistry confirmed normalization of renal values (creatinine 0.54 mg/dL; reference range 0.5–1.4 mg/dL; urea 21 mg/dL; reference range 16–56 mg/dL), resolving the transient azotemia recorded on postoperative day 5; a mildly elevated alkaline phosphatase (218 U/L; reference range 1–114 U/L) was considered consistent with ongoing skeletal growth in this young dog, with alanine aminotransferase remaining within reference limits. No recurrence of the septic process was identified. The parallel temporal evolution of these clinical, laboratory, and bacteriological parameters over the hospitalization is illustrated in an integrated trend chart (Figure 8).

## 4. Discussion

### 4.1. Strengths

This case report combines several complementary diagnostic modalities—abdominal radiography, serial abdominal ultrasonography, quantitative bacterial culture on three postoperative occasions, antimicrobial susceptibility testing, histopathological examination, and macroscopic photographic documentation of the excised abscess—providing a comprehensive, multimodal characterization of the disease process from presentation through resolution. A particular strength of this report is the serial quantitative monitoring of bacterial load in the peritoneal exudate, an approach that has rarely been documented in the veterinary literature [12,16] and that offers an objective, reproducible measure of treatment response beyond qualitative cytological assessment alone. The diagnosis was further substantiated by clear histopathological confirmation of a mesenteric abscess with marked neutrophilic infiltration, and the temporal correlation observed between the declining bacterial counts, the resolution of clinicopathological abnormalities, and the sonographic regression of the abscess strengthens the internal consistency of the reported findings and supports their interpretation as genuine markers of clinical recovery.

### 4.2. Limitations

As with any single case report, the findings described here cannot be generalized, and no internal comparator group was available to contextualize the observed rate of bacterial clearance or clinical recovery. Advanced cross-sectional imaging (computed tomography) was not performed, which may have provided additional information on the extent of the abscess and its relationship to adjacent mesenteric structures. Established canine acute-phase inflammatory biomarkers—C-reactive protein (CRP) and serum amyloid A (SAA)—were not measured serially. We acknowledge that these are core indices for evaluating the systemic inflammatory component of sepsis recovery, and their absence represents a limitation of the present objective monitoring. Serial quantitative bacterial culture of the peritoneal exudate was nevertheless selected as the primary objective endpoint because it provides a direct, reproducible measure of the infectious burden at the source of peritonitis, whereas CRP and SAA are indirect systemic markers influenced by non-infectious confounders such as surgical tissue trauma and perioperative stress; serial leukocyte monitoring and clinical parameters provided complementary inflammatory and clinical tracking. Future studies should incorporate serial CRP/SAA alongside quantitative bacterial load to provide a combined microbiological and inflammatory assessment of sepsis recovery. Although direct Gram-stained microscopic examination of intraoperatively collected peritoneal fluid supported septic suppurative inflammation, peritoneal-fluid biochemical analysis was not performed; this limits comparison with standard cytological and biochemical criteria used to support the diagnosis of septic peritonitis and is acknowledged as a diagnostic limitation of the case. Specifically, only a Gram-stained smear was performed on the intraoperatively collected peritoneal fluid (also used for culture and susceptibility testing); the serial quantitative bacterial cultures were performed on separate drain samples collected from the abdominal drain tube on POD1–POD3 (see Section 3.4). Neither the intraoperative fluid nor the drain samples underwent formal cytological examination (total nucleated cell count and differential) or peritoneal-fluid biochemical analysis (glucose, lactate, total protein). Furthermore, while the temporal relationship between the preceding babesiosis and the development of the mesenteric abscess is noteworthy, a causal link cannot be established from this single case, and the association remains coincidental; nevertheless, a transient functional immunosuppression associated with babesiosis—characterized by reduced CD4+ and CD8+ T-lymphocyte populations and suppressed lymphocyte blastogenesis—has been documented in dogs [24], and could theoretically predispose to bacterial translocation and abscess formation, although this mechanism remains speculative in the present case. Additionally, serial quantitative bacterial culture was limited to three postoperative assessments, providing only a restricted temporal resolution of the decline in peritoneal bacterial load; the timing of these assessments was not standardized to a predefined sampling protocol. Because surgical source control, peritoneal lavage, open-drainage management, and antimicrobial therapy were applied concurrently, their individual contributions to the observed reduction in bacterial load cannot be separated. The case does not demonstrate a prognostic value for serial quantitative bacterial culture, and no validated CFU/mL thresholds currently exist to guide drain removal or antimicrobial duration; accordingly, the role of quantitative bacterial culture as a complementary monitoring strategy and its potential to guide these decisions prospectively remain to be evaluated in future studies.

### 4.3. Comparison with Literature

The present case adds to a very limited body of literature on mesenteric abscessation and purulent lymphadenitis in dogs, a condition previously described almost exclusively through isolated case reports [3,4] and a single larger retrospective series of only 14 dogs in which no underlying cause could be identified in any case [5]. In agreement with the isolates recovered in the present case (*Escherichia coli* and *Enterococcus faecium*), *Escherichia coli*, *Staphylococcus* spp., and, rarely, *Salmonella* spp. have been the most frequently reported bacterial isolates in canine and feline mesenteric lymphadenitis and abscessation [3,4,5,6]. These enteric organisms are members of the resident intestinal microbiota, the early establishment and composition of which in young animals can be shaped by diet and early microbial exposure [25]; when mucosal or lymphatic barriers are disrupted, such commensal bacteria may translocate and act as opportunistic pathogens, giving rise to mesenteric abscessation and secondary septic peritonitis.

Once the abscess ruptures and contaminates the peritoneal cavity, the prognosis becomes considerably more guarded: secondary septic peritonitis is recognized as the most common and most life-threatening form of peritonitis in small animal practice [8,9,10], with reported survival rates ranging from approximately 36% to 85% and with advanced age, elevated lactate, and postoperative hypoalbuminemia identified as negative prognostic factors [8,9,11]. The favorable outcome documented in this case—full clinical recovery confirmed at one-month follow-up—is therefore consistent with, but toward the more favorable end of, this previously reported range, and may reflect the combination of prompt surgical source control, susceptibility-guided antimicrobial therapy, and close postoperative monitoring.

Direct quantitative monitoring of bacterial load within the peritoneal cavity, as performed serially in this dog on three postoperative occasions, has rarely been reported in veterinary medicine; to date, only a small number of studies have evaluated sequential bacterial culture as an objective marker of treatment response after surgical and antimicrobial management of septic peritonitis [12,16]. It should be emphasized that TGC quantifies viable bacterial burden (CFU/mL), whereas peritoneal-fluid cytology characterizes the inflammatory cell population and identifies intracellular bacteria; the two methods therefore provide complementary, non-interchangeable information. Although a direct microscopic examination of the peritoneal fluid was performed by Gram-stained smear (Figure 1, documenting intracellular bacteria and polymorphonuclear cells), a formal cytological examination (total nucleated cell count and differential) was not performed (see Section 4.2); consequently, TGC cannot be compared with cytological criteria and is not presented as a substitute for cytology. The progressive decline in bacterial counts observed in this case, paralleling clinical and hematological improvement, is consistent with a potential complementary monitoring role for this approach. However, whether quantitative bacterial culture can prospectively guide antimicrobial duration and the timing of drain removal remains to be established in future studies, as these decisions were not based on predefined CFU/mL thresholds. In the present case, the drain was removed after three postoperative days, once serial TGC had shown a consistent decline (from 3.12 to 4.3 × 10^5^ CFU/mL on POD1 to 2.8 × 10^4^ CFU/mL on POD3), and this was accompanied by clinical improvement (normothermia and return of appetite) and hematological improvement; drain removal was therefore based on combined microbiological, clinical, and hematological criteria rather than on the bacterial count alone.

Serial abdominal ultrasonography, used here both for the initial diagnosis and for postoperative monitoring, is central to the diagnostic work-up of intra-abdominal abscessation and septic peritonitis, although its sensitivity for correctly identifying the underlying lesion remains imperfect, ranging from approximately 56% to 87% across recent series [13,14,15]. In this case, ultrasonography successfully identified the mesenteric abscess at presentation and subsequently documented its progressive resolution, illustrating its practical value as a repeatable, non-invasive monitoring tool when used alongside other diagnostic modalities.

### 4.4. Antimicrobial Therapy and Resistance Considerations

Empirical perioperative antimicrobial therapy comprised amoxicillin–clavulanate (Synulox^®^, 20 mg/kg SC q24h) and metronidazole, a combination intended to provide broad coverage of Enterobacterales and anaerobes. This regimen reflects recommended first-line empirical therapy for suspected polymicrobial septic peritonitis in dogs; no local antibiogram data were available at the time of presentation to refine the empirical selection, which was therefore based on the expected spectrum of causative pathogens (Enterobacterales and anaerobes) rather than on local resistance patterns. However, susceptibility testing subsequently demonstrated that the *Escherichia coli* isolate was resistant to amoxicillin–clavulanate (as well as to ampicillin, cefuroxime, doxycycline, and chloramphenicol), retaining susceptibility only to enrofloxacin and meropenem; the empirical regimen therefore provided inadequate coverage against the Enterobacterales component of the infection. This highlights the limited predictive value of empirical therapy in the absence of local antibiogram data and underscores the importance of early source control and rapid transition to targeted therapy once susceptibility results become available.

The *Enterococcus faecium* isolate was multidrug-resistant, susceptible only to chloramphenicol and resistant to ampicillin, doxycycline, vancomycin, and enrofloxacin. This profile is consistent with the characteristic resistance of *E. faecium*, which commonly exhibits reduced susceptibility to aminopenicillins through low-affinity penicillin-binding proteins and frequent resistance to fluoroquinolones; the addition of clavulanate does not reliably restore activity against enterococci, as their resistance is not primarily beta-lactamase-mediated. Although a causal link cannot be established in a single case, it is plausible that selection pressure from the four-day preoperative amoxicillin–clavulanate course favored overgrowth of intrinsically resistant enterococci by suppressing competing susceptible flora, thereby facilitating enterococcal translocation across the disrupted mucosa of the fistulized abscess. Equally, *E. faecium* may have been a primary translocating pathogen from the intestinal tract rather than a de novo-selected resistant organism.

Beyond the immediate antimicrobial considerations, intestinal dysbiosis and the associated systemic inflammatory response are increasingly recognized as contributing to the pathophysiology of septic peritonitis, and microbiota-targeted modulation—including postbiotic-based interventions shown to alter intestinal microbial composition and inflammatory parameters—remains an area of active investigation as a potential complementary strategy [26].

The subsequent susceptibility-guided combination of enrofloxacin (active against *E. coli*) and chloramphenicol (active against *E. faecium*) achieved microbiological and clinical resolution, illustrating the value of prompt susceptibility testing and de-escalation and the need for local resistance surveillance to inform rational empirical therapy. Although chloramphenicol carries a recognized, dose-dependent risk of bone-marrow suppression in dogs, this risk was mitigated by the short 7-day subcutaneous course and by serial complete-blood-count monitoring throughout hospitalization; the platelet count, markedly reduced at presentation (52 × 10^9^/L), progressively normalized to 469 × 10^9^/L during treatment, providing no evidence of chloramphenicol-induced myelosuppression, and the benefit of treating a multidrug-resistant *E. faecium* infection with limited therapeutic alternatives was judged to outweigh this risk.

### 4.5. Lessons Learned

This case underscores the value of a multimodal diagnostic approach—combining diagnostic imaging, bacteriological culture with susceptibility testing, and histopathological examination—for establishing a definitive diagnosis of mesenteric abscessation and septic peritonitis in dogs. It further illustrates the practical value of serial quantitative bacterial culture as an objective method for documenting treatment response; whether it can prospectively guide antimicrobial duration and drain removal remains to be evaluated in future studies.

## 5. Conclusions

This case suggests that a multimodal diagnostic approach—combining serial abdominal ultrasonography, quantitative bacterial load monitoring, antimicrobial susceptibility testing, and histopathological examination—may assist in guiding the management of canine mesenteric abscess complicated by septic peritonitis and provide objective confirmation of treatment response. The serial decline in peritoneal bacterial counts paralleled clinical and hematological improvement, supporting a potential complementary monitoring role for quantitative bacterial culture that warrants prospective evaluation. Complete recovery in this patient indicates that surgical source control combined with susceptibility-guided antimicrobial therapy and perioperative supportive care was associated with a favorable outcome, and this observation warrants further investigation in larger series of dogs with septic peritonitis.

## 6. Patient/Owner Perspective

The owner first noticed that the dog had stopped eating and began vomiting, together with clear signs of abdominal discomfort, over a 72 h period, and recalled that the dog had recently completed treatment for babesiosis. The diagnosis of a septic abdominal infection and the need for surgery were considered frightening; however, the owner reported feeling reassured by the daily updates on the dog’s laboratory results and bacterial counts during hospitalization. At the one-month recheck, the owner described the dog as fully recovered, active, and back to its normal behavior, and expressed satisfaction with the multimodal monitoring approach used throughout treatment.

## Data Availability

The original contributions presented in this study are included in the article/Supplementary Material. Further inquiries can be directed to the corresponding author.

## Supplementary Materials

The following supporting information can be downloaded at: https://www.mdpi.com/article/10.3390/vetsci13090946/s1, Table S1: Timeline of principal clinical events and serial monitoring during the 11-day hospitalization.

## Abbreviations

AST	Antimicrobial Susceptibility Testing
CARE	CAse REport (Reporting Guidelines)
CFU	Colony-Forming Unit
CLSI	Clinical and Laboratory Standards Institute
HEA	Hematoxylin–Eosin–Azure
MALDI-TOF	Matrix-Assisted Laser Desorption/Ionization–Time of Flight
TGC	Total Germ Count
TSA	Tryptic Soy Agar
POD	Postoperative Day
