# Supplementary material for: Mesenteric Abscess Complicated by Septic Peritonitis in a Dog: Multimodal Diagnosis, Surgical Management, and Serial Quantitative Bacterial Load Monitoring
Source: Vet Sci. 2026 Sep 11;13(9):946. doi: 10.3390/vetsci13090946 (PMC13612059; doi:10.3390/vetsci13090946)
Supplement: Supplementary file 1 [file vetsci-13-00946-s001.zip › vetsci-4543398-supplementary.pdf]

Supplementary Table S1. Timeline of principal clinical events and serial monitoring during the 11-day hospitalization.

Day (POD)	Clinical events and monitoring
**Day 1**	Presentation/admission: abdominal pain, vomiting, anorexia; T 39.5 °C, HR 100 bpm. WBC 26.6 $\times 10^9$/L, platelets 52 $\times 10^9$/L, creatinine 0.51 mg/dL, urea 42 mg/dL. Admission ultrasonography: distended small-intestinal loops, accelerated peristalsis, linear hyperechoic intraluminal foreign body, small volume of free peritoneal fluid. Empirical antimicrobial therapy started (metronidazole + amoxicillin–clavulanate); fluid therapy; butylscopolamine.
**Day 2**	Exploratory laparotomy: en bloc resection of the mesenteric abscess; peritoneal lavage; abdominal drain placement; bacteriological sampling of the abscess contents. T 40.8 °C, HR 108 bpm. Creatinine 0.54 mg/dL, urea 29 mg/dL. Tranexamic acid added prophylactically (thrombocytopenia, mild anemia).
**Day 3 (POD1)**	First quantitative peritoneal culture (TGC): 3.12–4.3 $\times 10^5$ CFU/mL. WBC peak 56.09 $\times 10^9$/L. Normothermic (38.6–38.8 °C). Postoperative analgesia started (methadone, meloxicam). Abdominal drain in situ. Creatinine 0.53 mg/dL, urea 28 mg/dL.
**Day 4 (POD2)**	Second TGC: 2.31–2.6 $\times 10^5$ CFU/mL. Antimicrobial susceptibility results available → switch to susceptibility-guided therapy: enrofloxacin + chloramphenicol (7-day course, Days 4–11). Creatinine 0.44 mg/dL, urea 29 mg/dL.
**Day 5 (POD3)**	Third TGC: 2.8 $\times 10^4$ CFU/mL. Abdominal drain removed (maintained POD1–POD3). Appetite returned; normothermic (38.8 °C).
**Day 7 (POD5)**	Ultrasonographic reassessment: reduced loop distension, normalized peristalsis, resolution of invagination tendency, reduced free peritoneal fluid. Creatinine 1.20 mg/dL, urea 16 mg/dL.
**Day 11**	Discharge: clinically stable, normal appetite and vital parameters. Platelets 469 $\times 10^9$/L. Susceptibility-guided antimicrobial course completed.

TGC, total viable (aerobic) bacterial count, expressed as CFU/mL; WBC, white blood cell count; POD, postoperative day; HR, heart rate. Reference intervals: WBC 6–17 $\times 10^9$/L; platelets 165–500 $\times 10^9$/L; creatinine 0.5–1.4 mg/dL; urea 16–56 mg/dL.
